# The Nuclear Landscape: A 3D Map of Human Chromosomes

**DOI:** 10.1371/journal.pbio.0030188

**Published:** 2005-04-26

On this, theologians, philosophers, and biologists can agree: we are more than the sum of our genes. Biological complexity arises not from gene number but from patterns of gene expression, which change under the direction of both genetic and so-called epigenetic mechanisms. Epigenetics, broadly defined, concerns heritable changes in gene function that don't involve changes in DNA sequence. Until recently, studies of heritable traits have focused largely on mutations in DNA. But it's become increasingly clear that how DNA is packaged in the nucleus also impacts heritability.

Epigenetic changes are mediated largely by proteins that shape and remodel chromatin—the association of DNA and histone proteins that condenses the genome into compact bundles inside the nucleus. Different cell types have different chromatin arrangements during development and cell differentiation that appear to regulate gene expression, which possibly accounts for the unique gene expression patterns associated with specific cell types. Such phenomena have been well-studied for specific genes or chromosomal regions, but to understand the full impact of epigenetic mechanisms on gene regulation, we need a more panoramic view of gene organization within the nucleus.

In a new study, Thomas Cremer together with Andreas Bolzer and an interdisciplinary team of German physicists, bioinformaticians, and geneticists created 3D positional maps of each human chromosome simultaneously in a single nucleus to investigate the link between chromatin structure and cell-specific gene expression. Working with human fibroblasts, cultured from a skin biopsy from a two-year-old boy, the authors were able to visualize and study the order of the full genetic complement within a human nucleus.[Fig pbio-0030188-g001]

**Figure pbio-0030188-g001:**
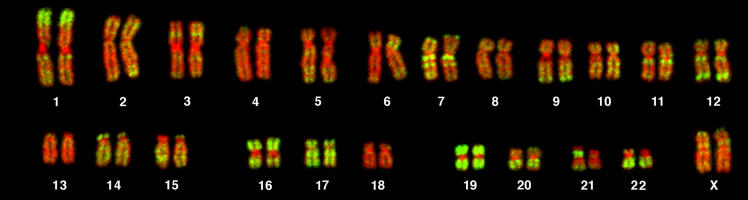
In this karotype from a female human lymphocyte, the gene-rich areas are stained green and the gene-poor areas are red (Photo: Irina Solovei)

Cremer and colleagues first produced a 3D topological map of all 46 chromosomes in different cell types at key points in the cell cycle—a landmark achievement—using a fluorescent staining technique that preserves chromosome shape during visual inspection under the microscope. Next, they established that small chromosomes in quiescent (nondividing) fibroblasts hewed close to the center of the nucleus while the large chromosomes were preferentially found at the nuclear rim, regardless of their gene density. Nuclei from cells entering the prometaphase stage of the cell cycle—just before chromosomes are aligned along the center of the nucleus prior to segregation—revealed a size-correlated chromosomal distribution akin to that seen in the quiescent nuclei. Statistical modeling analyses indicated that these size correlations do not simply reflect the geometric constraints of fitting into the nucleus, but likely hint at some degree of functional order within the nucleus.

Because previous studies of cells with sphere-like nuclei correlated chromosomal arrangements with gene density, the authors investigated how shape affects chromosome position along the nuclear radius. Fibroblast nuclei are somewhat flat and ellipsoidal. Chromosomes in similarly shaped amniotic fluid cells assumed the same size-related positions taken by chromosomes in fibroblast nuclei. But when the authors examined the higher-order chromatin arrangements in fibroblasts and lymphocytes, they found that, even though the cell types differ in nuclear shape and radial chromosomal arrangements, they both show a nonrandom higher-order chromatin architecture correlated with gene density. Many questions remain concerning the functional and physiological significance of these observations: Do shape changes produce changes in chromosomal arrangements and vice versa? Do shape changes produce changes in gene expression patterns?

Cremer and colleagues conclude that, although nonrandom chromosome positions occur, these appear to be governed by a degree of uncertainty and more likely reflect probabilistic preferences inside the nucleus. Still, deterministic mechanisms in higher-order chromatin structure may exist—sequestering gene-rich chromatin areas in the nuclear interior, for example, protected from malevolent agents entering the nucleus. And given the coexistence of size-correlated features with gene-density-correlated features seen in this study, it may well be that both random and deterministic factors combine to create the nuclear landscape.

